# Cooperative Search and Rescue with Artificial Fishes Based on Fish-Swarm Algorithm for Underwater Wireless Sensor Networks

**DOI:** 10.1155/2014/145306

**Published:** 2014-03-05

**Authors:** Wei Zhao, Zhenmin Tang, Yuwang Yang, Lei Wang, Shaohua Lan

**Affiliations:** Computer Department, Nanjing University of Science and Technology, Jiangsu 210094, China

## Abstract

This paper presents a searching control approach for cooperating mobile sensor networks. We use a density function to represent the frequency of distress signals issued by victims. The mobile nodes' moving in mission space is similar to the behaviors of fish-swarm in water. So, we take the mobile node as artificial fish node and define its operations by a probabilistic model over a limited range. A fish-swarm based algorithm is designed requiring local information at each fish node and maximizing the joint detection probabilities of distress signals. Optimization of formation is also considered for the searching control approach and is optimized by fish-swarm algorithm. Simulation results include two schemes: preset route and random walks, and it is showed that the control scheme has adaptive and effective properties.

## 1. Introduction

Recently, with the development of wireless sensor networks, especially the mobile wireless sensor networks, more and more applications emerge and have received general attention. Examples include pollution detection, wildfire monitoring, search and rescue missions, reconnaissance, and surveillance. In this paper, we position the application background at the problem of underwater search and rescue [1–3]. As we all know, underwater search and rescue is a very dangerous activity which requires professionals to conduct, and the rescue workers generally need to receive professional training to do this job. The primary task of search and rescue is to find and position the victim, and due to the complicated underwater environment, this is a very difficult job. With the development of bionics, the emergence of artificial fish [4–6] maybe has provided us with a cooperative search and rescue plan. We can install the searching device onto the artificial fish, and when the fish find the victim, they will send the position signal to the rescuing ship. In addition, through coordinated searching by multiple artificial fishes, the probability to find the victim can be significantly increased, and in the meantime, the searching time can be shortened, which is vital in rescuing tasks. This paper studies the problem of how to coordinate multiple artificial fishes and conduct efficient and timely search, and it will be a meaningful task to discuss this problem.

The problem proposed in this paper is similar to the fundamental problem of cooperative coverage control or active sensing [7–10]. Cooperative control refers to settings which involve multiple controllable agents cooperating toward a common objective. In [7], the authors propose a coverage control algorithm aimed at maximizing target exposure in some surveillance applications, while in [11–14], heuristic algorithms based on potential fields and virtual forces are applied to push nodes away from each other and disperse them into the unoccupied areas in the mission space to enhance the coverage of a sensor network. The potential fields and virtual forces approach imitates the behavior of electromagnetic particles: when two electromagnetic particles are too close in proximity, a repulsive force pushes them apart. Applied to a sensor network, this method helps move sensors from high-density to low-density areas, thereby minimizing sensing overlap and improving the overall network coverage. In [8], a decentralized coverage control algorithm is proposed based on centroidal Voronoi partitioning, and a dynamic version of the Lloyd algorithm [15] has been used to iteratively find such a configuration. Other related works based on Voronoi's partitions are included in [16, 17]. However, Lloyd's method suffers from two critical issues when it is used in mobile sensor networks. First, it does not consider the limited sensor communication range. Secondly, it does not optimize sensor movement distance; hence, it can lead to excessive energy consumption, a primary concern in sensor networks. Much of the active sensing literature [10] also concentrates on the problem of tracking specific targets using mobile sensors and the Kalman filter is extensively used to process observations and generate estimates.

In this paper, we consider a setting which involves a team of fish nodes and a set of target points (victims) in a three-dimensional space (e.g., under water). Each target point represents a victim. A mission is defined as the process of controlling the movement of the fish nodes and ultimately assigning them to target points so as to find the victims by visiting points within a given mission time *T*. In [18], the authors consider a setting where multiple vehicles form a team cooperating to visit multiple target points and collect rewards associated with them. Related work was proposed in their papers [19, 20]. Different from the papers mentioned above, in this paper, the target points are totally unknown in advance, or just located in an approximate range area. In addition, we pay more attention to find the target points in the shortest time. Because the earlier the victim can be found, the less the losses there will be; thus, we propose a cooperative coverage control scheme aiming at finding the target points in the shortest time under an uncertain environment.

The rest part of this paper is organized as follows: in Section 2, we propose the distributed cooperative searching control scheme for underwater mobile sensor networks. In Section 3, we optimize the scheme mentioned in Section 2 by fish-swarm algorithm. In Section 4, we simulate the distributed cooperative searching control algorithm by using computer software and evaluate its performance. Finally, in Section 5, we reach the main conclusions.

## 2. Fish-Swarm Searching Trajectory Model

**Figure 1:**
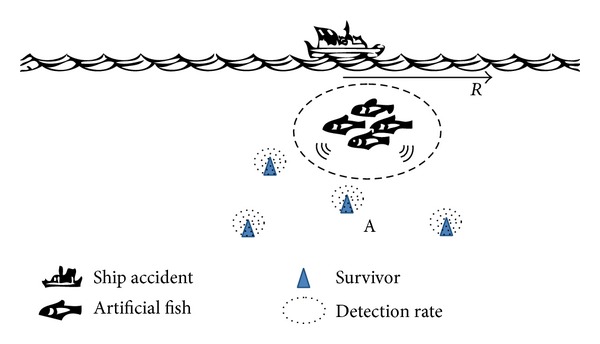
Task scenario description.

We imagine a classic scenario: consider Area *A* with *C*(*x*, *y*) as its center of circle, and it has a radius of *R* and a depth of *d*. This could be a case of accident in the ocean, the accident center is the circle center *C*, the survivors may be scattered in Area *A* with a radius of *R*, but their specific locations are unknown, and the task objective is to find all the survivors in Area *A* with no omission, as shown in Figure 1.

**Figure 2:**
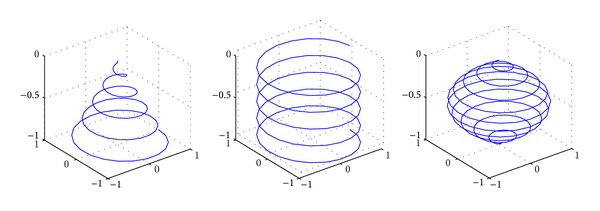
Helix curve.

Under the actual situation, the survivors tend to be scattered around the accident center, and the further from the center, the less the survivors there will be. In this paper, the mobile nodes participating in the search task are assumed as the fish swarm, and based on the situation mentioned above, the following two schemes are considered: in one scheme, after the fish swarm (mobile nodes) reaches the accident center, random walk is adopted; in another scheme, cruise is conducted in accordance with a certain preset route. The so-called random walk refers to that the fish swarm moves toward a certain direction; when they reach the task boundary, they randomly choose another direction and continue swimming, but the whole swimming track has to be within the task Area *A*. Here, we will discuss the second scheme, and in the experiment part of this paper, the effects of the two schemes will be compared. Assume the trajectory equation of fish swarm's cruise is *F*(*r*, *θ*, *z*, *t*), which represents that at any moment *t*, the spatial position of fish swarm is (*r*, *θ*, *z*), and Figure 2 has listed the corresponding helixes of several alternative helix equations. Of course, this is only an assumption, any equation will work, and the only difference is the effect.

In accordance with Figure 2, we can see that none of these helix curves satisfies the searching requirement; that is, there is a blind spot in exploration. An ideal track of fish swarm should satisfy the following several conditions. Firstly, it should cover the whole Area *A*. Secondly, it only needs to search any position in the area once; that is, there is repeated area, and in this way, it can ensure the shortest searching time. Thirdly, the trajectory equation should not be too complicated, which will sabotage the mechanical realization of robot fish's route. Therefore, in accordance with the above conditions, this paper constructs an applicable helix equation, which can be expressed as the following in a polar coordinate system:

(1) F(r,θ,z,t) ={r=RT(t−2nT),   2nT≤t<(2n+1)T,  n=0,1,2,…,r=−RT(t−2nT−T)+R,     (2n+1)T≤t<(2n+2)T,  n=0,1,…,θ=θ0+R2Rs2πt,z=z0−2Rsm,     mT≤t≤(m+1)T,  m=0,1,2,….

**Figure 3:**
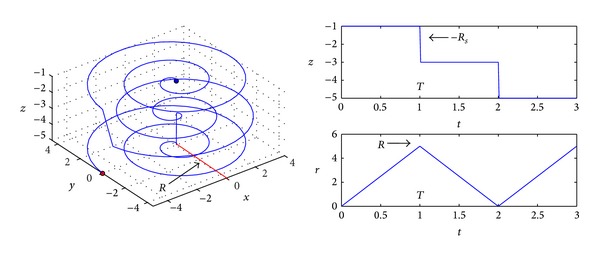
Helix constructed in this paper.

In Formula (1), *R* refers the search radius of Area *A*, *T* is a search cycle, the whole search process consists of multiple search cycles *T*, *R*_*s*_ is the perceived radius of node, *θ*_0_ and *z*_0_ represent the angle and the initial position of axis *z*, respectively, and *m* and *n* represent integers. In accordance with the left diagram in Figure 3, the node moves from the initial position (blue dot) to the final position (red dot) through the helix, during which, 3 cycles (*T*) of search have been conducted, the search of each cycle represents a plane on axis *z*, and it gets deeper and deeper. Therefore, the trajectory can cover the whole Area *A*, and there is no repeated path.

In accordance with the above right diagram in Figure 3, this is the curve of the search depth of fish swarm changing with time, the initial altitude of search is decided by *z*_0_, and here, *z*_0_ = −*R*_*s*_, which can just cover the sea surface. After a search cycle *T* is completed, the fish swarm needs to search in a deeper position; that is, *z*_0_ − 2*R*_*s*_, and in this way, it can ensure that there will not be any omission or repeated search area. We call the search plane at the same altitude completed in each cycle *T* a search layer, and the whole search process consists of multiple such search layers. The lower part of the right diagram in Figure 3 is the curve of search radius *r* changing with time. The initial position is at the accident center, so *r* = 0; as the fish swarm diffuses to the surrounding area, *r* reaches the maximum search radius *R* in a search layer; then, it will conduct backward search in the next search later, and *r* gradually reduces from *R* to 0. The benefits of trajectory equation with such design include full coverage of Area *A* and there is no area with repeated search, and in the meantime, the fish swarm can smoothly transit between various search layers.

## 3. Optimization Formation with Fish-Swarm Algorithm

If only one artificial fish participates into the search task, there is no need for formation optimization. Of course, during the actual process, in order to increase the searching scale and shorten the searching time, it is more reasonable to adopt collaborative search. Therefore, it will generate the formation optimization problem of fish swarm. Apparently, the formation of searching fish swarm should satisfy the following two requirements: firstly, the search section of fish swarm should be as big as possible; secondly, the fish swarm should maintain connection; that is, they should be within the communication range. Naturally, it is appropriate for us to use the artificial fish swarm optimization algorithm (AFSA) [21, 22] to optimize the formation of fish swarm.

### 3.1. Network Coverage

First of all, let us discuss the calculation of network coverage. As an important index to measure the strategy of sensor network deployment, the network coverage is generally defined as the ratio between the whole area that can be covered by nodes in the monitored area and the total monitored area. Considering the complication of monitoring environment in actual application, this paper has adopted the probability measurement model in the literature [23] to calculate the network coverage. Assume the total number of nodes is *N*, *s*_*i*_ representing the *i*th node in the network; then, the corresponding node set is *S* = {*s*_*i*_ | *i* = 1,2,…, *N*}. Assume *p*(*x*, *y*, *z*) is a random point in Area *A*, *p* ∈ *A*; then the distance between node *s*_*i*_(*xi*, *yi*, *zi*) and point *P* is

(2) $$\begin{array}{c} D\left(s_{i},p\right)=||s_{i}-p||=\sqrt{{\left(x_{i}-x\right)}^{2}+{\left(y_{i}-y\right)}^{2}+{\left(z_{i}-z\right)}^{2}}. \end{array}$$

By adopting the probability measurement model in the literature [23], the detection probability of node *s*_*i*_ to point *p* is

(3) $$\begin{array}{c} P_{p}\left(s_{i}\right)=\{\begin{array}{cc} 0, & R_{s}+R_{e}\leq D\left(s_{i},p\right)\\ e^{-\alpha \lambda^{\beta }}, & R_{s}-R_{e}<D\left(s_{i},p\right)<R_{s}+R_{e}\\ 1, & R_{s}-R_{e}\geq D\left(s_{i},p\right), \end{array} \end{array}$$

in which *R*_*s*_ refers to the perceived radius of various nodes in the network, *R*_*e*_ refers to the uncertain factors within the measurement range of nodes, and 0 < *R*_*e*_ < *R*_*s*_; *α* and *β* refer to the measured parameters related to the physical device; *λ* is the input parameter, which is defined as

(4) $$\begin{array}{c} \lambda =D\left(s_{i},p\right)-\left(R_{s}-R_{e}\right). \end{array}$$

Therefore, we can obtain the joint detection probability of multiple sensor nodes simultaneously conducting measurement to the target point *p* as

(5) $$\begin{array}{c} P_{p}\left(S\right)=1-\prod_{n_{i}\in B}\left(1-P_{p}\left(s_{i}\right)\right), \end{array}$$

in which *S* refers to the sensor node set of the measured target point. Therefore, in order to realize full coverage of the target area, it requires satisfying the following condition:

(6) $$\begin{array}{c} \min\left\{P_{p}\left(S\right)\right\}\geq c_{\text{th}}, \end{array}$$

in which *c*_th_ (0 < *c*_th_ < 1) refers to the threshold value of detection probability set in accordance with different application requirements. In order to calculate the coverage of sensor network, we need to conduct grid processing of the monitored area (the bigger the distance between adjacent grid points is, the higher the calculation accuracy is), and then, the joint detection probability at each grid point is solved. The percentage of grids which meet *P* ≥ *c*_th_ is called network coverage.

**Figure 4:**
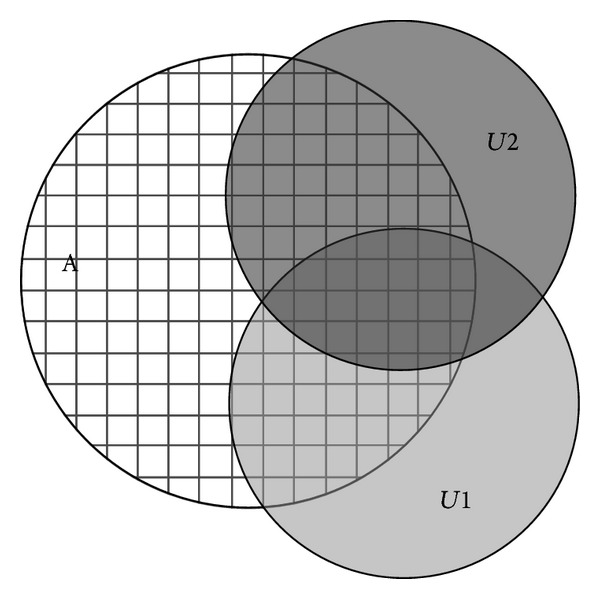
Schematic diagram of network coverage.

As shown in Figure 4, Area *A* with grids is the monitored area, and the shadow areas *U*1 and *U*2 represent the perceived areas of *s*_1_ and *s*_2_, respectively. Assume Area *A* is divided into *N* grids, and *p* refers to a random grid; then its network coverage is

(7) $$\begin{array}{c} \frac{N\left\{\min\left\{P_{p\in (A\cap (U1\cup U2))}\left(s_{1},s_{2}\right)\right\}\geq c_{\text{th}}\right\}}{N}. \end{array}$$

**Figure 5:**
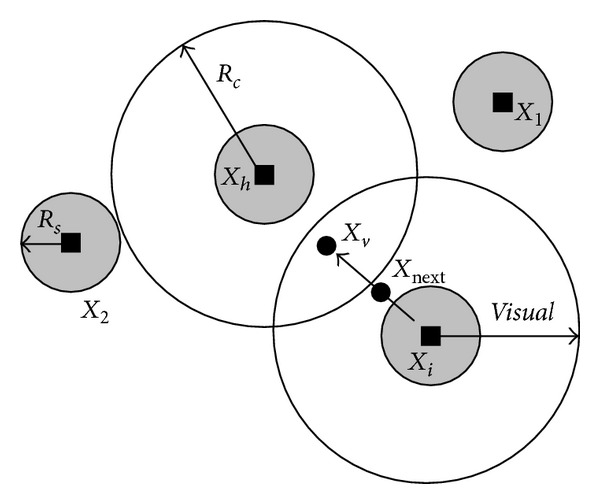
Vision concept of the artificial fish.

In Formula (7), the numerator refers to the number of grids that satisfy the threshold value of detection probability *c*_th_, while the denominator refers to the total number of grids. Because our task model is in a 3-dimensional space, the calculation process is conducted in accordance with the 3D grids, and Figure 5 only shows an example.

Assume under current state, the position vector of all sensor nodes in the network is *U* = [*X*_*n*_ *Y*_*n*_ *Z*_*n*_], and the function to calculate the network coverage with *U* as the input variable is *f*(*U*), in which *X*_*n*_ = {*x*_1_, *x*_2_,…, *x*_*n*_}, *Y*_*n*_ = {*y*_1_, *y*_2_,…, *y*_*n*_}, and *Z*_*n*_ = {*z*_1_, *z*_2_,…, *z*_*n*_} are the node coordinate vectors. We abstract the optimization of network layout into solving the optimization problem with *f*(*U*) as the objective function.

### 3.2. Optimization with Fish-Swarm Algorithm

The artificial fish-swarm optimization algorithm (AFSA) is an optimization algorithm which simulates the behavior of fish swarm, and it uses the preying, gathering, and chasing behavior of fish swarm to find fast and global optimum solution. In this paper, the fish swarm consisting of mobile nodes can also be regarded as a cluster of nodes, in which the cluster head node (the leader of fish swarm) conducts cruise in accordance with the trajectory equation *F*(*r*, *θ*, *z*, *t*) mentioned in the last section; other nodes are processed in accordance with the fish swarm algorithm, and in this way, it can ensure that the whole fish swarm maintains reliable communication and a maximum search scale.

Assume in an *n*-dimensional target search space, there is a fish swarm consisting of *N* artificial fish, and every day, the state of individual artificial fish can be expressed as vector **X** = (*x*_1_, *x*_2_,…, *x*_*n*_), in which *x*_*i*_ (*i* = 1,…, *n*) is the variable that needs to be optimized: the food concentration of current location where the artificial fish are can be expressed as *Y* = *f*(**X**), in which *Y* is the objective function; the distance between individual artificial fish can be expressed as *d*_*ij*_ = ||**X**_*i*_ − **X**_*j*_||; visual refers to the perceived range of artificial fish, step is the moving step length of artificial fish, and *δ* is the crowding degree factor; try_number represents the maximum trying times each time the artificial fish go preying.

The AF realizes external perception by its vision shown in Figure 5.  *X*_*i*_ is the current position of an AF, *X*_*h*_ is the cluster head's position, *Visual* (*R*_*c*_, communication radius) is the visual distance, and *X*_*v*_ is the visual position at some moment. If the position at the visual position is better than the current position, it goes forward a step in this direction, and arrives at the *X*_next_ position; otherwise, it continues an inspecting tour in the vision.

In the sensor network, the process of mobile nodes exploring toward bigger network coverage is similar to the chasing and preying behavior by individual artificial fish, and the food concentration of current location where the artificial fish are can be regarded as the network coverage under current state. Fish usually stay in the place with a lot of food, so we simulate the behaviors of fish based on this characteristic to find the global optimum, which is the basic idea of the AFSA. The basic behaviors of AF are defined as follows.

*(1) AF_Prey.* This is a basic biological behavior heading for the food; generally the fish perceives the concentration of food in water to determine the movement by vision or sense and then chooses the tendency.

Behavior description: let *X*_*i*_ be the AF current state and select a state *X*_*j*_ randomly in its visual distance, *Y* is the food concentration (objective function value), the greater the *Visual* is, the more easily the AF finds the global extreme value and converges:

(8) $$\begin{array}{c} X_{j}=X_{i}+Visual.Rand\left(\;\right). \end{array}$$

If *Y*_*i*_ < *Y*_*j*_ in the maximum problem, it goes forward a step in this direction; otherwise, select a state *X*_*j*_ randomly again and judge whether it satisfies the forward condition. If it cannot satisfy after try_number times, it moves a step randomly. When the try_number is small in AF_Prey, the AF can swim randomly, which makes it flee from the local extreme value field:

(9) $$\begin{array}{c} X_{i}^{\left(t+1\right)}=X_{i}^{\left(t\right)}+Visual.Rand\left(\;\right). \end{array}$$

*(2) AF_Swarm. *The fish will assemble in groups naturally in the moving process, which is a kind of living habits in order to guarantee the existence of the colony and avoid dangers. Behavior description: let *X*_*i*_ be the AF current state, *X*_*c*_ the center position, and nf the number of its companions in the current neighborhood (*d*_*ij*_ < *Visual*), *N* is the number of total fishes. If *Y*_*c*_ > *Y*_*i*_ and *nf*/*N* < *δ*, which means that the companion center has more food (higher fitness function value) and is not very crowded, it goes forward a step to the companion center:

(10) $$\begin{array}{c} X_{i}^{\left(t+1\right)}=X_{i}^{\left(t\right)}+\frac{X_{c}-X_{i}^{\left(t\right)}}{||X_{c}-X_{i}^{\left(t\right)}||}Step.Rand\left(\;\right). \end{array}$$

Otherwise, it executes the preying behavior. The crowd factor limits the scale of swarms, and more AF only cluster at the optimal area, which ensures that AF moves to optimum in a wide field.

*(3) AF_Follow.* In the moving process of the fish swarm, when a single fish or several ones find food, the neighborhood partners will trail and reach the food quickly. Behavior description: let *X*_*i*_ be the AF current state, and it explores the companion *X*_*j*_ in the neighborhood (*d*_*ij*_ < *Visual*), which has the greatest *Y*_*j*_. If *Y*_*j*_ > *Y*_*i*_ and *nf*/*N* < *δ*, which means that the companion *X*_*j*_ state has higher food concentration (higher fitness function value) and the surroundings is not very crowded, it goes forward a step to the companion *X*_*j*_:

(11) $$\begin{array}{c} X_{i}^{\left(t+1\right)}=X_{i}^{\left(t\right)}+\frac{X_{j}-X_{i}^{\left(t\right)}}{||X_{j}-X_{i}^{\left(t\right)}||}Step.Rand\left(\;\right). \end{array}$$

Otherwise, it executes the preying behavior.

*(4) AF_Move.* Fish swim randomly in water; in fact, they are seeking food or companions in larger ranges. Behavior description: it chooses a state at random in the vision; then it moves towards this state; in fact, it is a default behavior of AF_Prey:

(12) $$\begin{array}{c} X_{i}^{\left(t+1\right)}=X_{i}^{\left(t\right)}+Visual.Rand\left(\;\right). \end{array}$$

Therefore, by using the gathering and chasing behavior of fish swarm, it can draw the nodes close to the cluster head. In the meantime, in order to realize maximum search range, the fish swarm should maintain a good formation, which could be controlled through the crowding factor *δ* and the distance between fishes. The formation optimization process based on artificial fish swarm consists of the following specific steps.

- Initialize the wireless sensor network. Ensure the scale of artificial fish swarm *N* in accordance with the application requirement, the maximum moving step length of artificial fish is *step*, the visible range of artificial fish is *visual*, the maximum iteration times are *k*, and the crowding degree factor is *δ*.
- Initialize the fish swarm *X*, randomly generate *N* individual artificial fish within the task space, and in the meantime, set the initial iteration times as *k* = 0.
- Calculate the food concentration of current location where the initial individual fish in the swarm is *Y*_*i*_  (i.e., the network coverage *Y*_*i*_); then put them in sequence, and select the individual artificial fish with the biggest value of *Y*_*i*_ to enter the billboard *T*.
- The artificial fish simulate the gathering and chasing behavior of fish swarm, and the fish with big *Y* value is selected to conduct preying behavior.
- After each action of various artificial fish, the food concentration of current location *Y* will be compared to the *Y*_*T*_ of artificial fish on the billboard, and if it is bigger than the value of *Y*_*T*_ on the Billboard, this artificial fish will replace the billboard fish and enter billboard.
- Determine the end condition. If it has reached the maximum iteration times, the *Y* value of billboard will be output, that is, the optimum formation solution; otherwise, *k* = *k* + 1, go to (d).

### 3.3. Example for Optimization Formation

**Figure 6:**
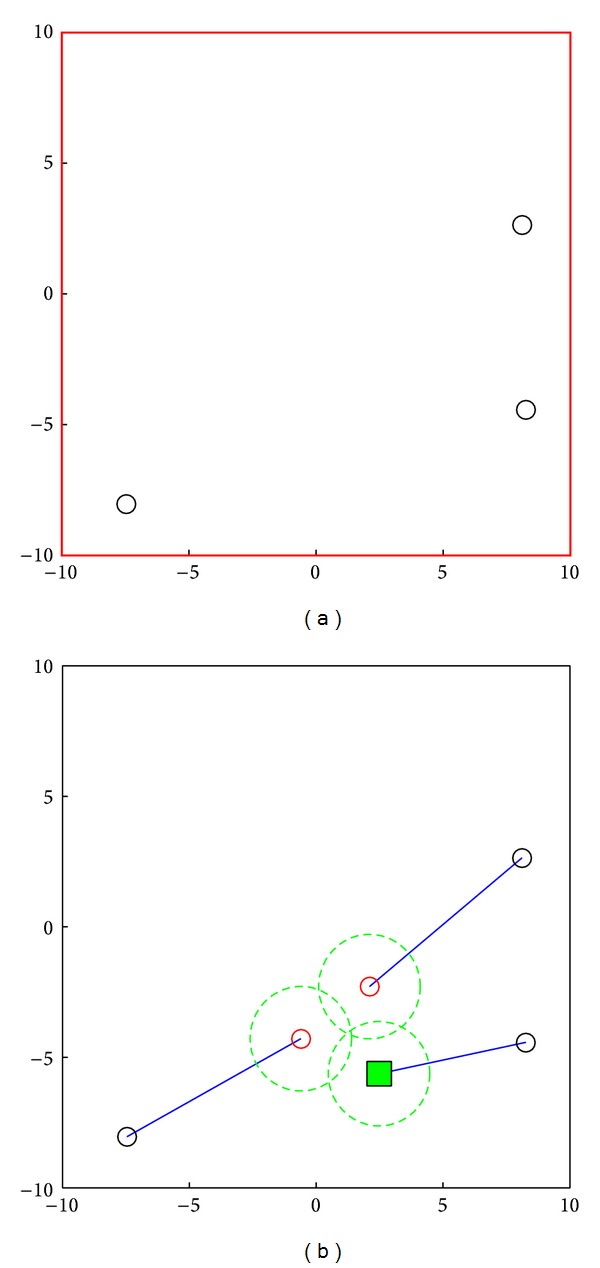
Example of 3 fishes forming a swarm.

How to make the fish swarm search in a bigger scale? An easy approach is to make all fish in the fish swarm stay on the same plane, this plane is vertical to the direction which this fish swarm moves toward, and this could realize maximization of the cross-section area. In the meantime, efforts should be made to avoid any gap in the middle of fish swarm because the gap might cause blind spot during the search. Take a search fish swarm of 3 fishes for example, and after the 3 fishes reach the accident center, respectively, the formation process will successively begin. The initial positions of the 3 fishes could be random, they are optimized in accordance with the optimization algorithm in Section 3.2, they come closer to form a fish swarm, in the meantime, the distance between individuals is controlled to avoid any gap, and Figure 6 has shown this process.

The small circles on the left diagram represent the initial positions of 3 fishes, the red small circles on the right diagram represent the final positions after forming a swarm, the blue solid line refers to the trace of moving from the initial position to the final position, and the green block represents the leader of fish swarm after formation (cluster head node).

**Figure 7:**
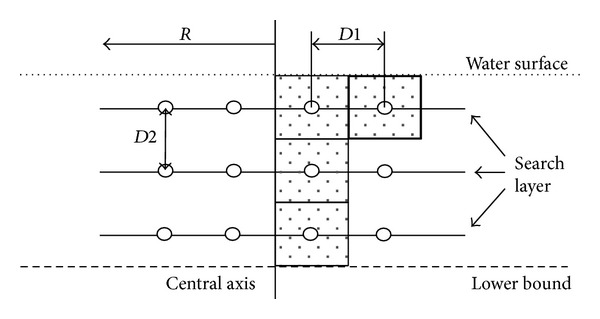
Diagrammatic sketch of search cross-section.

Apparently, if the search is conducted in accordance with the formation in Figure 6, it might cause blind spot during search because it is very difficult to ensure that there is no gap between various search layers. If there is a fish swarm consisting of *N* fish, for the convenience of not leaving any gap between various search layers or between pitches, as shown in Figure 7, we need to calculate the maximum rectangular cross-sectional area for searching the fish swarm.

**Figure 8:**
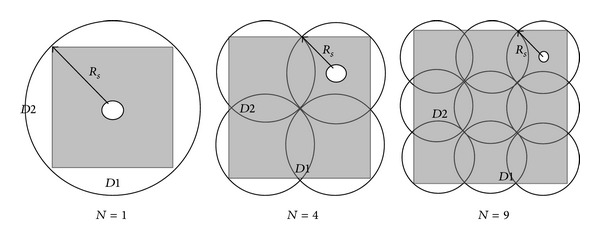
Diagrammatic sketches of *D*1 and *D*2 when *N* = 1,4, 9.

In Figure 7, *R* represents the radius of search area *A*, *D*1 refers to the space between helixes on the same search layer, and *D*2 refers to the space between various search layers. The shadow block area in bold represents the search cross-section, and we can see the size of cross-section is *D*1 × *D*2. When *N* = 1,4, 9, *D*1 and *D*2 are as shown in Figure 8.

## 4. Simulation and Results

We consider mobile nodes to be operating in a three-dimensional underwater mission space. Assume that the mission is to find survivors from *M* targets using *N* nodes. Let set *G* denote the *M* targets *G* = {*m*_*j*_ | *j* = 1,2,…, *M*} and let set *B* denote *N* nodes *B* = {*n*_*i*_ | *i* = 1,2,…, *N*}. Note that a target may change its location during operation of the system, or new targets may show up; hence *M* and *y*_*j*_ may not be constant. At the same time, a node may malfunction, and thus *N* may also change in time. Associated with the *j*th target is a life value *L*_*j*_, if *L*_*j*_ ≤ 0, this means target *j*th fails, and in reality, it might mean that the survivor is dead. The mission's objective is to maximize the total life value collected by visiting target points in the set *G* within a given mission time *T*. Target life value may be time dependent, typically decreasing in time. The exact location of targets may not always be known in advance and there may be obstacles in the mission space, which constrain the feasible trajectories of nodes.

We model the mission space as a cylinder *A* ⊂ **R** ^3^, over which there is an event density function *ρ*(*x*), *x* ∈ *A*, that captures the frequency or density that a specific event takes place (in Hz/m^3^). *ρ*(*x*) satisfies *ρ*(*x*) ≥ 0 for all *x* ∈ *A*. In this paper, *ρ*(*x*) may be the frequency that a survivor appears at target point. When an event occurs at point *x*, it emits a signal and this signal is observed by a sensor at that location nearby.

To distinguish the relative importance of targets at time *t*, each target has an associated life function denoted by *L*_*j*_Φ_*j*_(*t*), where *L*_*j*_ is the maximal life value and Φ_*j*_(*t*)∈[0,1] is a discounting function which describes the life value change over time. When a deadline is associated with a particular target point, we can use

(13) $$\begin{array}{c} \Phi_{j}\left(t\right)=\{\begin{array}{cc} 1-\frac{\mu_{j}}{H_{j}}t, & \;t\leq H_{j},\\ \left(1-\varepsilon_{j}\right)e^{-\eta_{j}(t-H_{j})}, & \;t>H_{j}, \end{array} \end{array}$$

where *H*_*j*_ is a deadline assigned to target point *j* and *ε*_*j*_ ∈ (0,1], *η*_*j*_ > 0 are parameters which may be target specific and are chosen to reflect different cases of interest.

The optimal searching problem can be formulated as an optimization problem to maximize the expected life value collected by the sensors over the mission space *A*:

(14) $$\begin{array}{c} \text{MAX}\int_{A}^{}L_{j}\Phi_{j}\left(A\right)\rho \left(A\right)P_{A}\left(B\right)F\left(A\right)dA. \end{array}$$

By referring to the above definition, *L*_*j*_Φ_*j*_(*A*) in Formula (14) refers to the fact that the life value represented by the target points in area *A* reduces with the discounting function, *ρ*(*A*)*P*_*A*_(*B*) refers to the joint detection probability of the target points in area *A* by all nodes in set *B*, and *F*(*A*) refers to the node trajectory equation in area *A*. Therefore, the meaning of Formula (14) is to maximize the expected life value collected by the sensors over the mission space *A*.

Table 1 shows the parameters of simulation experiment, and in order to make the experiment as close to the actual situation as possible, we have set the experiment parameters.

### 4.1. Total Search Time versus Nodes Number

**Figure 9:**
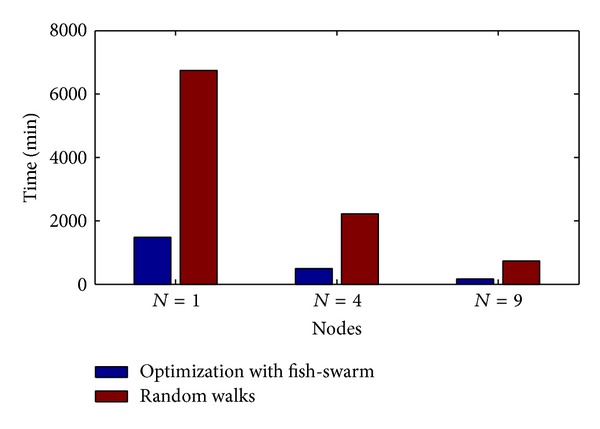
Overall searching time versus node number.

Figure 9 shows the relational diagram between the overall searching time and the node number under two schemes. In the first scheme, the fish swarm conducts cruise in accordance with a helix equation (blue bar graph), and in the second scheme, random walk is adopted. In accordance with the diagram, we can see that in both schemes, the overall searching time reduces with the increase of node number (*N* = 1,4, 9), but the random walk scheme takes significant more overall searching time than the helix scheme. When the node number is *N* = 1,4, 9, the time taken by the helix scheme is, respectively, 21.9%, 22.2%, and 22.6% of the time taken by the random walk scheme. When the node number is *N* = 1, the time taken by the helix scheme is 2.99 times of that when *N* = 4 and 8.96 times of that when *N* = 9. It can significantly reduce the searching time by increasing the number of fishes in the fish swarm, but of course, this will also increase the communication consumption and complexity. A feasible method is to increase the number of fish swarms, which can also achieve the effect of reducing the overall searching time.

### 4.2. Average Rescue Time versus Nodes Number

**Figure 10:**
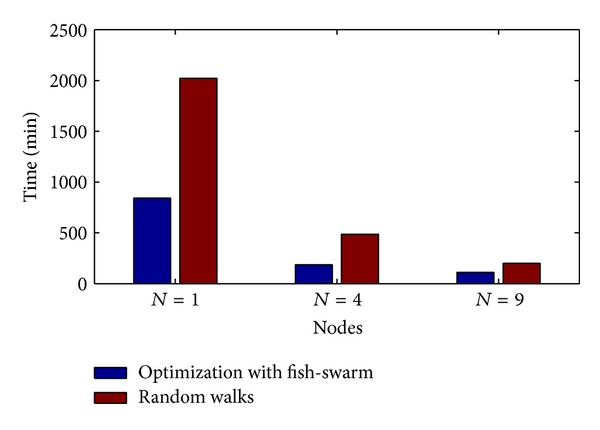
Average rescuing time versus node number.

Figure 10 shows the relational diagram between the average rescuing time and the node number under two schemes. The average rescuing time refers to the average time to find 5 target points. In accordance with the diagram, we can see that in both schemes, the average rescuing time reduces with the increase of node number, but the average rescuing time taken by the random walk scheme is longer than the helix scheme. When the node number is *N* = 1,4, 9, the time taken by the helix scheme is, respectively, 41.6%, 38.2%, and 55.4% of the time taken by the random walk scheme. In accordance with the set parameters in Table 1, when the node number is *N* = 1, the average rescuing time taken by the helix scheme is 841.5 minutes, and when *N* = 4 and *N* = 9, it takes 185.5 minutes and 111.2 minutes, respectively. This is far from the 10-minute threshold value of life value we expect, and the solutions include increasing the swimming speed of fish swarm, increasing the number of fish swarms, or increasing the number of fishes in the fish swarm. Increasing the swimming speed of fish requires consideration on the aspect of mechanics, which exceeds the discussion in this paper; increasing the number of fishes in the fish swarm is restricted because excessive nodes in the swarm will cause communication delay, which will also increase the control overhead. A feasible method is to increase the number of fish swarms while at the time ensuring that there will not be excessive fishes in the fish swarm, which will significantly reduce the average rescuing time.

### 4.3. Target Discovery Time

**Figure 11:**
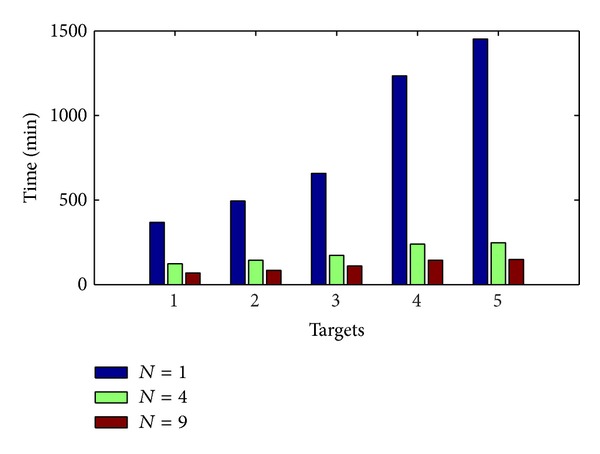
Target discovery time.

Figure 11 shows the target discovery time by the fish swarm in accordance with the helix equation under the cruise scheme, and 5 total target points are found, which are randomly distributed in the whole task Area *A*. Record the 5 time points where the fish swarm find them in accordance with the cruise route. In accordance with the diagram, we can see that the discovery time of 5 targets presents gradual progressive increase, which is easy to understand. In reality, the sooner the target is discovered, the more beneficial it is to the rescue work, which requires the target discovery time to be as short as possible. In the diagram, we can see that it can significantly reduce the target discovery time by increasing the node number. For example, when *N* = 9, the time to discover the first target is 244.9 minutes shorter than that when *N* = 1, and the time to discover the fifth target is 1204.2 minutes shorter. In the meantime, in order to further reduce the target discovery time, the number of fish swarms can also be increased, so that the fish swarms can conduct searching at different search layers, which can significantly reduce the target discovery time and increase the rescuing efficiency.

### 4.4. Optimized Formation versus No Optimization

**Figure 12:**
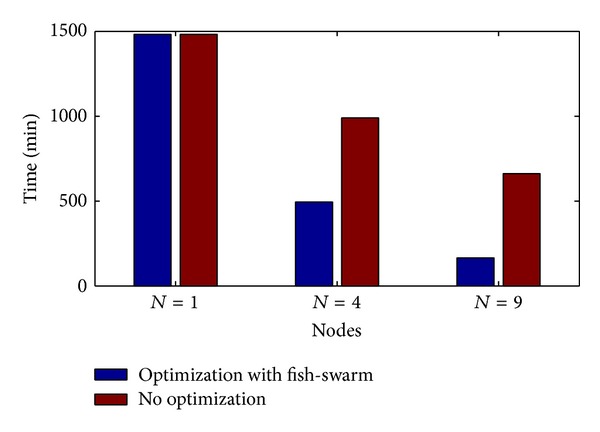
Comparison between the optimized formation and the formation with no optimization.

In accordance with the above result, we can see that the optimized formation can increase the search cross-sectional area of fish swarm, and Figure 12 shows the comparison between the optimized formation and the formation with no optimization when the node number is *N* = 1,4, 9. In accordance with the diagram, we can see that optimized formation to the fish swarm can significantly reduce the searching time. When *N* = 1, because there is only one fish, there is no so-called formation optimization; when *N* = 4, the efficiency has increased by 51.3% after optimization; when *N* = 9, the efficiency has increased by 74.6% after optimization. Apparently, the more nodes there are, the more significant the optimization result is. However, during the actual application, there should not be too many fishes in the fish swarm because this will cause a high cost of optimization.

## 5. Conclusions

Under the background of sea rescue, this paper proposes a search scheme based on the fish-swarm optimization algorithm, and the sensor nodes moving underwater are considered as the artificial fishes. In this paper, a trajectory model of fish swarm cruise is built in accordance with the helix equation, the fish-swarm optimization algorithm is used to optimize the formation of nodes, and the fish swarm behavior, such as gathering, chasing, and preying, is used to control the movement of nodes, so that maximum cross-sectional area of search by the fish swarm can be achieved. The experiment result shows that compared to the random walk model, it can significantly reduce the searching time in accordance with the preset trajectory cruise, which has reference value for practical application; in the meantime, it can also help reduce the searching time through formation optimization of fish swarm. Sea rescue is a task that has high requirement of saving time, the next step of work should be further in-depth research on the issues discussed in this paper, and how to adapt to underwater barriers will also be discussed.

## Figures and Tables

**Table 1:** Experiment parameters.

Network configuration	Node	Artificial fish	Others
Radius *R* = 1 km	*R*_*s*_ = 50 m	Swimming speed *V* = 1.5 m/s	Life value = 10 minutes
Depth *d* = 200 m	*R*_*c*_ = 100 m	*Visual* = *R*_*c*_	
*c*_th_ = 0.9	*R*_*e*_ = 0.5 *R*_s_	*Step* = *R*_*s*_	
*N* = {1,4, 9}	*α* = 0.2; *β* = 2.0		
Targets: *M* = 5			
